# HbA1c and Cognitive Functioning Across Bipolar Disorder, Recurrent Major Depressive Disorder, and Schizophrenia: Findings from a Non-Diabetic Sample

**DOI:** 10.3390/brainsci16070770

**Published:** 2026-07-22

**Authors:** Ece Buyuksandalyaci Tunc, Serhat Tunc, Murat Ilhan Atagun, Samet Kose

**Affiliations:** 1 Department of Psychiatry and Behavioral Neuroscience, School of Medicine, Saint Louis University, St. Louis, MO 63104, USA; dr.ecetunc@gmail.com; 2 Department of Psychiatry, Faculty of Medicine, Yeditepe University, Istanbul 34755, Türkiye; drserhattunc@gmail.com; 3 Department of Psychiatry, Faculty of Medicine, Canakkale Onsekiz Mart University, Canakkale 17110, Türkiye; muratilhanatagun@gmail.com; 4 Department of Psychiatry and Behavioral Sciences, Cam Sakura City Hospital, Health Sciences University, Istanbul 34295, Türkiye

**Keywords:** bipolar disorder, schizophrenia, cognitive impairment, HbA1c, metabolic dysregulation

## Abstract

**Highlights:**

**What are the main findings?**

- HbA1c levels were most strongly and consistently associated with poorer cognitive performance in bipolar disorder (BD), across multiple domains including processing speed, attention, executive functioning, memory, and social cognition, even within the non-diabetic range. In contrast, this association was weaker and limited in recurrent major depressive disorder (rMDD), and was absent in schizophrenia (SZ) despite SZ having the highest HbA1c levels among all groups.
- Antipsychotic exposure (CEDA) was associated with higher HbA1c levels and partially attenuated the HbA1c-cognition relationship, but did not fully account for the observed associations, indicating that the metabolic-cognitive link is not solely medication-driven and that pharmacological and metabolic factors may represent partially independent pathways contributing to cognitive dysfunction.

**What are the implications of the main findings?**

- Subclinical glycemic dysregulation may represent an underrecognized and potentially modifiable contributor to cognitive dysfunction in mood disorders, particularly BD. HbA1c, as a stable, practical, and widely available metabolic marker, could serve as a clinical indicator of cognitive vulnerability in psychiatric populations even in the absence of diabetes, warranting routine metabolic monitoring as part of cognitive assessment in these patients.
- The disorder-specific pattern of HbA1c–cognition associations suggests that different pathophysiological mechanisms drive cognitive impairment across psychiatric diagnoses. In BD, insulin resistance and chronic low-grade inflammation may synergistically impair cognition through glycemic pathways, whereas in SZ, neurodevelopmental and illness-related factors may predominate, potentially creating a floor effect that obscures metabolic contributions. This highlights the need for longitudinal studies to determine whether targeted metabolic interventions (e.g., insulin sensitizers, lifestyle modifications) could improve cognitive outcomes, particularly in BD.

**Abstract:**

**Objective:** This study examined the association between glycemic status, indexed by HbA1c, and cognitive functioning in individuals with bipolar disorder (BD), recurrent major depressive disorder (rMDD), schizophrenia (SZ), and healthy controls (HCs). **Methods:** In this cross-sectional study, 215 participants (45 HCs, 64 BD, 62 rMDD, 44 SZ) were assessed. Cognitive performance was evaluated using a comprehensive neuropsychological battery measuring attention, processing speed, memory, executive functions, and social cognition. Sociodemographic characteristics, chlorpromazine-equivalent antipsychotic doses (CEDA), and metabolic variables (body mass index, waist circumference, lipid profile, fasting glucose, and HbA1c) were collected. Group differences, correlation analyses, and multiple regression models were performed. **Results:** All patient groups showed significantly poorer cognitive performance compared with HCs. HbA1c levels were highest in the SZ group; however, associations between HbA1c and cognitive performance were strongest in the BD group and less consistent in rMDD. In regression analyses, HbA1c was associated with cognitive performance in BD and HCs, with weaker effects in rMDD. CEDA was associated with HbA1c levels and partially attenuated the relationship between HbA1c and cognitive outcomes. **Conclusions:** HbA1c levels were associated with cognitive performance in non-diabetic individuals, particularly in BD and rMDD. These findings suggest that subclinical variation in glycemic regulation may be relevant to cognitive functioning in psychiatric disorders. Further longitudinal studies are needed to clarify the influence of metabolic factors and psychotropic medication on cognitive outcomes.

## 1. Introduction

Bipolar disorder (BD), schizophrenia (SZ), and recurrent major depressive disorder (rMDD) are chronic and severe psychiatric disorders that affect millions of individuals worldwide and are associated with substantial functional impairment [1]. Beyond their core diagnostic symptoms, these disorders are characterized by significant cognitive dysfunction, which contributes to impairments in social and occupational functioning not only during acute episodes but also during periods of clinical stability [1,2,3]. Verbal memory, working memory, executive functioning, and attention are among the cognitive domains most consistently affected across these conditions [4]. Consequently, cognitive dysfunction has increasingly been recognized as a relatively independent psychopathological dimension that extends beyond traditional diagnostic boundaries [3].

Cognitive impairment has long been considered a core feature of SZ and is more strongly associated with functional outcomes than psychotic symptoms themselves [5]. Individuals with SZ exhibit deficits across multiple cognitive domains, including memory, attention, executive functioning, and processing speed [6,7,8]. In addition, impairments in social cognition, particularly theory of mind (ToM), have been consistently reported, even during periods of remission [9,10].

Similarly, cognitive dysfunction is a prominent feature of both BD and rMDD. Meta-analytic evidence has demonstrated impairments in attention, executive functioning, memory, and processing speed, with many of these deficits persisting during euthymic periods in BD [11]. Difficulties in ToM and other aspects of social cognition have also been documented in both BD and MDD, further contributing to functional impairment [12,13].

The mechanisms underlying cognitive dysfunction in psychiatric disorders are complex and likely involve the interaction of multiple biological pathways. Among these, metabolic dysregulation has emerged as a particularly important area of investigation. Cardiovascular and metabolic conditions, including hypertension, diabetes, metabolic syndrome, and hyperlipidemia, are known to adversely affect brain health and cognitive functioning. These effects may occur through several mechanisms, such as reduced cerebral blood flow, insulin resistance, chronic hyperglycemia, obesity, and dyslipidemia. Accordingly, metabolic health appears to play a critical role in maintaining cognitive functioning [14,15,16,17].

Medical comorbidity has been associated with a more severe course of BD. Previous studies have reported a high prevalence of insulin resistance and type 2 diabetes mellitus (DM) among individuals with BD, and insulin resistance may precede a more severe illness trajectory characterized by earlier onset and increased risk of relapse [18,19,20]. Furthermore, among adults without DM, the co-occurrence of depressive symptoms and metabolic syndrome has been linked to poorer cognitive performance [21]. Likewise, individuals with SZ appear to be predisposed to metabolic abnormalities from the early stages of illness [22], and these abnormalities have been associated with both cognitive impairment and disease progression [23].

Among metabolic markers, glycated hemoglobin (HbA1c) has received particular attention because it is a stable indicator of long-term glycemic exposure, reflecting average blood glucose levels over approximately 120 days. Unlike fasting glucose, insulin levels, or indices such as the homeostatic model assessment of insulin resistance (HOMA-IR), HbA1c is less influenced by short-term physiological fluctuations and does not require fasting, making it a practical and reliable measure of chronic glycemic status. Although an HbA1c value of 6.5% is widely accepted as the diagnostic threshold for DM, levels between 5.7% and 6.5% are considered indicative of increased risk [24]. Studies conducted in non-diabetic populations have demonstrated that higher HbA1c levels are associated with poorer cognitive performance, suggesting that even subclinical glycemic dysregulation may negatively affect brain function [25].

Several biological mechanisms have been proposed to explain the association between elevated HbA1c and cognitive dysfunction. Chronic hyperglycemia and insulin resistance have been associated with reduced cerebral glucose metabolism and impaired neuronal energy utilization [26]. Hyperglycemia and systemic insulin resistance also promote neuroinflammation, involving microglial activation and increased pro-inflammatory cytokine signaling across the blood–brain barrier [27]. In addition, chronic hyperglycemia increases the formation of advanced glycation end-products (AGE), which act via the AGE-RAGE (receptor for advanced glycation end-products) pathway to trigger oxidative stress and contribute to neuronal damage [28]. Elevated HbA1c has also been linked to endothelial dysfunction and cerebral microvascular impairment, which may reduce cerebral perfusion and contribute to white matter changes relevant to cognitive performance [29]. Notably, these associations extend to subclinical glycemic dysregulation, even in the absence of overt diabetes, with population-based studies linking higher HbA1c levels within the non-diabetic range to poorer cognitive outcomes and increased dementia risk [30]. These interrelated mechanisms provide a plausible biological basis for the association between subclinical glycemic dysregulation and cognitive impairment observed across the diagnostic groups in the present study.

However, a critical gap remains: despite growing evidence linking metabolic dysfunction to cognitive impairment, no study to date has directly compared the association between HbA1c and cognitive performance across multiple major psychiatric diagnostic categories, BD, rMDD, and SZ, within the same non-diabetic sample. To our knowledge, this is the first study to adopt this transdiagnostic approach, offering a novel perspective on the potential role of subclinical glycemic dysregulation in cognitive functioning across major psychiatric disorders.

Therefore, the present study aimed to compare cognitive functioning among individuals with SZ, BD, and rMDD and healthy controls (HCs), and to examine the association between HbA1c levels and cognitive performance in non-diabetic individuals with these disorders. We hypothesized that participants in the psychiatric groups would demonstrate poorer cognitive performance than HCs and that higher HbA1c levels, even within the non-diabetic range, would be associated with poorer performance across multiple cognitive domains.

## 2. Materials and Methods

### 2.1. Participants

This study included 64 patients with BD, 62 patients with rMDD, 44 patients with SZ, and 45 HCs. Patients were recruited consecutively between November 3, 2018, and March 31, 2019, from eligible individuals presenting to the Psychiatry Outpatient Clinic of Kafkas University Faculty of Medicine. All patients were clinically stable outpatients, evaluated at least 8 weeks after their last significant symptomatic episode, at the time of enrollment.

Clinical stability was assessed through evaluation by treating psychiatrists, supported by standardized rating scales. Depressive symptoms were assessed using the Hamilton Depression Rating Scale (HDRS), manic symptoms using the Young Mania Rating Scale (YMRS), and psychotic symptoms using the Scale for the Assessment of Positive Symptoms (SAPS) and the Scale for the Assessment of Negative Symptoms (SANS). Participants are described as clinically stable outpatients rather than as being strictly in remission, as a subset exceeded conventional symptom-severity thresholds on these scales despite being judged clinically stable by their treating psychiatrist.

HCs were recruited from among relatives of hospital staff and had no lifetime history of psychiatric disorders. All participants were between 18 and 65 years of age and had completed at least primary school education.

Exclusion criteria included a known diagnosis of diabetes mellitus (DM) or prediabetes, a history of neurodevelopmental disorders, neurocognitive disorders, hypertension, chronic metabolic or cardiovascular diseases, blood dyscrasias, hemoglobinopathies, chronic kidney disease, and any neurological condition potentially affecting cognitive performance. Current benzodiazepine use was also considered an exclusion criterion, given its known acute effects on cognitive performance, which could confound neuropsychological test results. HbA1c was measured as part of the study protocol to characterize glycemic status within the sample.

After providing written informed consent, all participants underwent a comprehensive psychiatric evaluation conducted by experienced psychiatrists. Diagnoses were confirmed according to the Diagnostic and Statistical Manual of Mental Disorders, Fifth Edition (DSM-5) criteria, based on clinical interviews and psychiatric evaluations conducted by experienced psychiatrists. Anthropometric measurements, including height, weight, and waist circumference, were obtained, and venous blood samples were collected for metabolic analyses. Participants subsequently completed a neuropsychological assessment administered by trained psychiatrists.

The study protocol was approved by the Kafkas University Faculty of Medicine Ethics Review Board (Approval No: 176; Date: 1 November 2018) and was conducted in accordance with the principles of the Declaration of Helsinki.

### 2.2. Laboratory Assessment

Blood samples were collected in EDTA tubes and analyzed for glycated hemoglobin (HbA1c) using an immunoturbidimetric inhibition assay on a Roche Cobas c501 analyzer (Roche Diagnostics GmbH, Mannheim, Germany) at the central biochemistry laboratory of Kafkas University Hospital. HbA1c values were expressed as a percentage of total hemoglobin.

### 2.3. Cognitive Tests

Cognitive functioning was evaluated using a battery of standardized neuropsychological tests assessing attention, executive functioning, memory, processing speed, and social cognition.

The Stroop Test was used to assess attention, inhibitory control, and cognitive flexibility, functions primarily associated with frontal lobe activity [31]. Its validity and reliability have been established in Turkish populations [32].

Theory of mind (ToM) abilities were assessed using the Dokuz Eylül Theory of Mind Scale (DEZTÖ), which evaluates several dimensions of ToM, including the understanding of beliefs, intentions, and social inferences. The scale has demonstrated adequate validity and reliability in Turkish populations [33].

The Trail Making Test (TMT) was administered to assess visual attention, processing speed, and cognitive flexibility [34].

The Digit Symbol Substitution Test (DSST), a subtest of the WAIS-R, was used to measure processing speed and visuomotor coordination [35].

The Digit Span Test was used to evaluate attention and short-term verbal memory, including both forward and backward components [36].

The Adult Memory and Information Processing Battery (AMIPB) was used to assess information-processing speed and working memory through timed number-comparison tasks [37].

Reaction time was measured using computerized visual and auditory stimuli presented across multiple trials.

### 2.4. Statistical Analysis

Statistical analyses were performed using SPSS version 24 (IBM Corp., Armonk, NY, USA). The normality of continuous variables was evaluated using the Kolmogorov–Smirnov test. Categorical variables were compared using the chi-square test.

Continuous variables were analyzed using the independent-samples t-test or Mann–Whitney U test for two-group comparisons and one-way analysis of variance (ANOVA) or the Kruskal–Wallis test for comparisons involving three or more groups, as appropriate. Homogeneity of variances was assessed using Levene’s test. Post hoc analyses were conducted using the Bonferroni correction when variances were homogeneous and Tamhane’s T2 test when the assumption of homogeneity was violated.

All statistical tests were two-tailed, and *p*-values <0.05 were considered statistically significant.

Hierarchical linear regression analyses were performed to examine the association between HbA1c levels and neurocognitive test performance. Variables known to influence cognitive functioning, including age, sex, education level, and body mass index (BMI), were entered in the first step of the models where applicable. HbA1c levels were entered in the subsequent step to evaluate their independent contribution to cognitive outcomes. Chlorpromazine-equivalent antipsychotic doses (CEDA) were entered in the final step to control for the potential confounding effects of antipsychotic treatment. Standardized regression coefficients (β), explained variance (R^2^), and changes in explained variance (ΔR^2^) were reported for all models.

Analyses were hypothesis-driven rather than exploratory, as the cognitive domains examined were selected a priori based on prior literature and the predefined objectives of the study; formal correction for multiple comparisons was therefore not applied.

Missing data across cognitive and metabolic variables ranged from approximately 0.9% to 5.1% and were handled using listwise deletion for the relevant analyses. CEDA data were available only for patients receiving antipsychotic treatment, consistent with its clinical applicability.

## 3. Results

### 3.1. Sociodemographic and Clinical Characteristics

No significant differences were found between groups in terms of age, gender, or years of education. However, significant group differences were found in clinical variables. Duration of illness was significantly longer in the SZ group compared with both BD and rMDD groups (BD < SZ, *p* = 0.008; rMDD < SZ, *p* < 0.001). Age at onset was significantly earlier in the SZ group compared with the rMDD group (*p* < 0.001). The number of hospitalizations was lower in the rMDD group compared with the other patient groups (*p* < 0.001). Antipsychotic exposure, as indexed by CEDA, was significantly higher in the SZ group compared with both BD and rMDD groups (*p* = 0.024 and *p* < 0.001, respectively). Sociodemographic and clinical characteristics are presented in Table 1.

### 3.2. Cognitive Performance

All patient groups demonstrated significantly poorer performance than HCs across multiple cognitive domains, including attention, executive functioning, processing speed, and memory. The most pronounced impairments were observed in the SZ group, particularly in processing speed (DSST), cognitive flexibility (TMT), and executive functioning (Stroop Test). The BD and rMDD groups also showed significant cognitive deficits compared with HCs; however, these impairments were generally less severe than those observed in SZ. Differences between BD and rMDD were less consistent across domains.

Notably, the absence of significant group differences in error rates on several measures (e.g., DSST errors) suggests that cognitive impairments were driven primarily by reduced processing speed rather than accuracy deficits (Table 2).

### 3.3. Metabolic Results

Significant group differences were observed in metabolic parameters (Table 3). The BD group showed higher BMI compared with HCs, whereas waist circumference was elevated in both BD and SZ groups. Low-density lipoprotein (LDL) and total cholesterol levels were higher in the rMDD group compared with HCs. HbA1c levels differed significantly across groups and were highest in the SZ group. Despite elevated HbA1c levels in SZ, associations between HbA1c and cognitive performance were limited in this group. Fasting glucose levels did not differ significantly between groups.

### 3.4. Association Between HbA1c and Cognitive Performance

HbA1c levels were significantly associated with multiple cognitive outcomes across groups (Table 4, Table 5 and Table 6). Correlation analyses revealed the most extensive associations in the BD group, where HbA1c was significantly correlated with TMT-A (*p* < 0.05), TMT-B (*p* < 0.01), AMIPB-A1/A2 (*p* < 0.01), AMIPB-B1/B2 (*p* < 0.01), ToM total score (*p* < 0.05), DSST (*p* < 0.01), Digit Span (*p* < 0.05), Digit Span backward (*p* < 0.05), and multiple Stroop duration and error indices (*p* < 0.05 to *p* < 0.01).

In the rMDD group, significant correlations were limited primarily to TMT-B (*p* < 0.05). In HCs, significant associations were observed for AMIPB-A1 (*p* = 0.037), AMIPB-B1 (*p* = 0.006), AMIPB-B2 (*p* = 0.012), DSST (*p* < 0.001), Digit Span (*p* = 0.039), and Stroop duration measures (ST-1 *p* = 0.006; ST-2 *p* < 0.001; ST-3 *p* < 0.001; ST-4 *p* < 0.001; ST-5 *p* = 0.019). In contrast, no significant correlations were observed in the SZ group despite the highest HbA1c levels (Table 4).

Regression analyses yielded a similar pattern (Table 5 and Table 6). In the BD group, HbA1c was associated with performance on multiple neurocognitive measures, including TMT-A (*p* = 0.002), TMT-B (*p* < 0.001), AMIPB-A1 (*p* = 0.008), AMIPB-B1 (*p* = 0.008), AMIPB-B2 (*p* = 0.003), ToM total score (*p* = 0.028), DSST (*p* = 0.002), Digit Span (*p* = 0.032), Digit Span backward (*p* = 0.005), and Stroop duration indices (ST-1 *p* = 0.003; ST-2 *p* < 0.001; ST-3 *p* = 0.029; ST-4 *p* < 0.001; ST-5 *p* = 0.021).

In the rMDD group, HbA1c was associated with only TMT-B performance (*p* = 0.035). In HCs, HbA1c was associated with AMIPB-A1 (*p* = 0.037), AMIPB-B1 (*p* = 0.006), AMIPB-B2 (*p* = 0.012), DSST (*p* < 0.001), Digit Span (*p* = 0.039), and Stroop duration measures (ST-1 *p* = 0.006; ST-2 *p* < 0.001; ST-3 *p* < 0.001; ST-4 *p* < 0.001; ST-5 *p* = 0.019). No significant regression effects were observed in the SZ group.

Prior to regression analyses, assumptions of linearity, normality of residuals, homoscedasticity, multicollinearity (VIF < 5), independence of residuals (Durbin–Watson ≈ 2), and influential observations (Cook’s distance < 1) were examined. All assumptions were satisfied.

### 3.5. Effects of Antipsychotic Treatment and Psychotropic Medication

Detailed information on psychotropic medication use across diagnostic groups, including CEDA, lithium and valproic acid serum levels, is provided in Supplementary Table S1. Hierarchical regression analyses examined the potential confounding effect of antipsychotic treatment on the association between HbA1c and cognitive performance. In the BD group, the association between HbA1c and AMIPB-A1 was attenuated and became non-significant after inclusion of CEDA in the second step of the model (F = 2.88, *p* = 0.067), suggesting a potential partial confounding effect of antipsychotic exposure. However, CEDA adjustment did not meaningfully alter the associations between HbA1c and other cognitive measures.

Additional analyses of psychotropic medications in the BD group showed that lithium use was associated only with TMT-B performance (Z = 198.00, *p* = 0.030), whereas valproate use was associated with ToM total score (t = 2.53, *p* = 0.022) and Digit Span performance (t = 2.06, *p* = 0.044). No other significant medication-related differences were observed.

## 4. Discussion

### 4.1. Cognitive Impairment Across Diagnostic Groups

In this cross-sectional study, we investigated the relationship between HbA1c levels and cognitive performance in individuals with SZ, BD, rMDD, and HCs without DM. Consistent with previous literature, we observed broad cognitive impairments across all three clinical groups, encompassing attention, processing speed, working memory, executive function, and theory of mind (ToM). Extending prior findings, our main contribution is demonstrating that HbA1c levels are associated with cognitive performance in BD, rMDD, and HCs, and that HbA1c is associated with multiple cognitive outcomes in BD and, to a lesser extent, in rMDD, even within the non-diabetic range. These findings suggest that subtle variations in glycemic regulation may contribute to cognitive heterogeneity, particularly in mood disorders, while playing a more limited role in SZ. Although antipsychotic exposure (CEDA) was associated with both higher HbA1c levels and poorer cognitive performance, adjustment for CEDA did not fully explain the observed associations, indicating at most a partial confounding effect. Overall, these results underscore the potential relevance of HbA1c as a marker of cognitive vulnerability, even in individuals without DM. To our knowledge, this is among the first studies to directly compare HbA1c–cognition associations across SZ, BD, rMDD, and HCs in a non-diabetic sample. Notably, the strength of this relationship varied across diagnostic groups, being most pronounced in BD, weaker in rMDD, and minimal in SZ despite the highest HbA1c levels in this group.

Consistent with extensive prior research, all patient groups demonstrated poorer cognitive performance than HCs across multiple domains. These impairments involved attention, processing speed, working memory, executive function, and social cognition, reflecting the multidimensional nature of cognitive dysfunction in psychiatric disorders [4,8,13]. Although cognitive deficits were present across all clinical groups, they were most pronounced in SZ, particularly in processing speed and executive functioning, in line with previous findings [7]. This pattern likely reflects illness-related factors such as earlier onset, longer illness duration, and neurodevelopmental contributions in SZ. Collectively, these results support the view that cognitive impairment in SZ is more pervasive and established than in mood disorders, even during clinically stable phases. At the same time, differences between BD and rMDD were less distinct than those reported in some previous comparative studies [38,39,40,41], possibly reflecting clinical heterogeneity, differences in remission criteria, or methodological variability across studies. Nevertheless, the significant impairments observed in both BD and rMDD relative to HCs reinforce the notion that cognitive dysfunction represents a transdiagnostic feature of psychiatric disorders rather than being restricted to psychotic conditions [3,42].

### 4.2. HbA1c and Cognitive Performance: Diagnostic Group Differences

Our findings indicate that higher HbA1c levels are associated with poorer cognitive performance in BD and, to a lesser extent, in rMDD, whereas these associations were largely absent in SZ despite elevated HbA1c levels. This pattern is partially consistent with prior studies suggesting that subclinical glycemic variation is linked to deficits in attention, processing speed, executive function, and memory in psychiatric populations [30,43,44,45]. 

### 4.3. HbA1c and Cognition in Schizophrenia

However, findings in SZ populations have been inconsistent. While some studies have reported significant associations between HbA1c and cognition, including executive function, attention/vigilance, and social cognition [43,46], our results did not replicate these associations in SZ. This discrepancy may be explained by differences in sample characteristics, as previous studies often included recent-onset or schizophrenia spectrum populations [43,46], whereas our SZ sample was characterized by longer illness duration and earlier onset, suggesting a more entrenched cognitive impairment less sensitive to metabolic variation. Methodological differences in cognitive assessment and analytic approaches may also contribute. Additionally, in SZ, cognitive dysfunction may be more strongly driven by neurodevelopmental and illness-related factors, potentially overshadowing the contribution of glycemic dysregulation. Additionally, a floor effect may be relevant: because cognitive performance in the SZ group was already substantially and globally impaired relative to other groups, the residual variance available to be explained by HbA1c may have been reduced, limiting the sensitivity of regression models to detect an association, even where one might exist biologically [47]. Although mechanisms such as insulin resistance, neuroinflammation, and endothelial dysfunction are biologically plausible contributors to cognitive impairment in SZ [48], their detection in this sample may have been obscured by the pervasive, illness-related cognitive burden already present in this group.

### 4.4. HbA1c and Cognition in Bipolar Disorder

The findings in BD are particularly noteworthy. Most prior research has examined broader metabolic constructs—such as insulin resistance, metabolic syndrome, obesity, or fasting glucose—and has consistently linked metabolic dysregulation in BD with poorer cognitive performance across domains including processing speed, attention, executive function, and memory [45,49,50,51,52]. However, direct evidence focusing specifically on HbA1c in BD is scarce. In this context, our results extend the literature by showing that HbA1c, a stable and clinically accessible marker of long-term glycemic regulation, is strongly associated with cognitive performance in BD, even in the absence of DM. This robust association may reflect the combined burden of insulin resistance and chronic low-grade inflammation, both of which have been independently implicated in BD pathophysiology, potentially acting synergistically with glycemic dysregulation to impair prefrontal and hippocampal circuits [53]. This suggests that subtle disturbances in glycemic control may represent an underrecognized contributor to cognitive dysfunction in BD. Importantly, these associations were most consistent in BD compared with the other groups, indicating a potential disorder-specific sensitivity to metabolic variation. Clinically, this raises the possibility that HbA1c may serve as a practical marker of cognitive vulnerability in BD, complementing broader metabolic indicators previously studied, although longitudinal studies are needed before HbA1c could be considered a validated predictive biomarker.

### 4.5. HbA1c and Cognition in Recurrent Major Depressive Disorder

Evidence in rMDD is comparatively limited, particularly in non-diabetic samples and in relation to HbA1c specifically [54,55]. The modest but significant associations observed in our rMDD group therefore extend the current literature by suggesting that glycemic variation may also contribute to cognitive vulnerability in depressive disorders. Previous research has more commonly focused on metabolic syndrome or insulin resistance rather than HbA1c as a specific marker. Indirect evidence has suggested bidirectional links among depressive symptoms, metabolic dysregulation, and cognitive deficits [56,57,58]. The comparatively weaker association observed here may reflect a less pronounced or more heterogeneous inflammatory and vascular burden in rMDD relative to BD, consistent with reports of lower rates of chronic low-grade inflammation in major depressive disorder compared with BD depression [59]. Although our rMDD sample was clinically stable, residual depressive symptoms were present in a subset of participants (see Limitations); nonetheless, clinical stability was confirmed by treating psychiatrists at the time of assessment, suggesting that the observed associations are unlikely to be solely attributable to acute symptom severity and may instead indicate a more stable relationship between metabolic status and cognitive functioning. Overall, these findings suggest that the HbA1c-cognition relationship may differ across psychiatric diagnoses and may reflect disorder-specific pathophysiological mechanisms.

### 4.6. HbA1c and Cognition in Healthy Controls

Interestingly, even in HCs, higher HbA1c levels were associated with poorer performance in attention, processing speed, and memory. This finding suggests that glycemic variation may influence cognitive functioning beyond clinical populations. It is consistent with evidence showing that HbA1c levels within the non-diabetic range are associated with poorer cognitive performance across multiple domains and age groups [30,44,60]. Longitudinal studies further indicate that higher HbA1c levels and greater glycemic variability are associated with accelerated cognitive decline, particularly in memory and executive functions [61,62,63]. These findings support a dose–response relationship between glycemic status and cognitive outcomes. Neuroimaging studies also provide converging evidence, linking higher HbA1c levels to reduced grey matter volume, altered white matter integrity, and smaller hippocampal volume [30,64], suggesting biological pathways through which glycemic variation may influence brain structure and function.

### 4.7. Effects of Antipsychotic Treatment

In our sample, the influence of CEDA on the HbA1c-cognition relationship was limited. Adjustment for CEDA attenuated the association between HbA1c and AMIPB-A1 only in the BD group, rendering it non-significant, while other associations remained largely unchanged. This suggests that antipsychotic exposure may contribute to specific cognitive outcomes but does not fully account for the overall HbA1c–cognition relationship. Although somewhat inconsistent with the broader literature suggesting that antipsychotics can impair cognition directly and indirectly via metabolic dysregulation [65,66,67,68], our findings suggest that the metabolic–cognitive link is not solely medication-driven.

One possible explanation is that CEDA effects were detectable only in BD, where HbA1c–cognition associations were strongest, whereas in groups with weaker associations (rMDD) or absent associations (SZ), additional variance attributable to antipsychotic dose was less apparent. Importantly, the literature examining the combined relationship among antipsychotic exposure, HbA1c, and cognition remains limited, and no prior study has simultaneously modeled these variables within a unified analytical framework. Accordingly, our findings suggest that while antipsychotic exposure may contribute to both metabolic and cognitive alterations, it does not fully explain the observed pattern. Instead, pharmacological and metabolic factors may represent partially independent, potentially interacting pathways contributing to cognitive dysfunction [43,46].

### 4.8. Limitations

Several limitations of this study should be acknowledged. First, the cross-sectional design precludes any causal inferences regarding the relationship between HbA1c levels and cognitive performance. Second, the relatively small sample size, particularly within diagnostic subgroups, may have reduced statistical power to detect more subtle associations. Relatedly, although the cognitive domains examined were selected a priori and analyses were hypothesis-driven, the number of statistical comparisons conducted increases the risk of Type I error, and findings should be interpreted with appropriate caution. Third, although antipsychotic exposure was accounted for using chlorpromazine-equivalent doses, the potential confounding effects of psychotropic polypharmacy, including mood stabilizers and antidepressants, could not be fully controlled, leaving the possibility of residual confounding. Fourth, we did not obtain direct measures of insulin resistance (e.g., HOMA-IR) or inflammatory markers, which limits our ability to disentangle the specific metabolic pathways underlying the observed HbA1c–cognition associations. Fifth, the absence of neuroimaging data precluded examination of the structural or functional brain correlates that may mediate this relationship. Sixth, unmeasured lifestyle factors (e.g., diet, physical activity, and smoking) and underlying neurobiological differences may have influenced the observed associations. Seventh, formal oral glucose tolerance testing was not performed; as a result, some participants with undiagnosed subclinical dysglycemia may have been included, as reflected in the HbA1c values observed in a subset of participants. Eighth, although participants were considered clinically stable outpatients by their treating psychiatrists, some exceeded conventional symptom-based remission thresholds on the HDRS, YMRS, SAPS, or SANS. Consequently, the potential influence of residual symptoms on cognitive outcomes cannot be excluded. Finally, given that the sample was drawn from a single clinical center in a specific geographic region, the generalisability of these findings to other populations, healthcare settings, or more ethnically and clinically diverse samples may be limited, and replication in multicenter, more heterogeneous cohorts is warranted.

## 5. Conclusions

In conclusion, the present findings suggest that HbA1c levels are associated with cognitive performance in individuals with BD, rMDD, and HCs, even in the absence of diabetes mellitus. These results indicate that subclinical variations in glycemic regulation may be relevant to cognitive functioning, particularly in mood disorders. However, the strength and pattern of these associations varied across diagnostic groups, being most robust and consistent in BD, comparatively weaker and less consistent in rMDD, and largely absent in SZ despite the highest HbA1c levels in this group; causal relationships cannot be inferred due to the cross-sectional design.

Accordingly, HbA1c should be interpreted as a potential marker of cognitive vulnerability rather than a direct or definitive predictor of cognitive decline. Overall, these findings underscore the importance of considering metabolic health in the assessment of cognitive dysfunction in psychiatric populations. Further longitudinal research is needed to clarify the complex interactions between glycemic regulation, psychotropic treatment, and cognitive outcomes, and to determine whether targeted metabolic interventions may contribute to cognitive improvement in these clinical groups.

## Figures and Tables

**Table 1:** Sociodemographic and clinical characteristics of the study groups.

		HC (*n* = 45)	BD (*n* = 64)	rMDD (*n* = 62)	SZ (*n* = 44)	F/X^2^	*p*
**Age**		33.07 ± 9.59	33.42 ± 11.18	36.95 ± 9.16	36.48 ± 10.69	2.04	0.109
**Gender (woman)**		24 (53.33%)	30 (46.88%)	39 (62.90%)	18 (40.91%)	6.35	0.096
**Marital Status (married)**		20 (44.44%)	34 (53.13%)	45 (72.58%)	9 (20.46%)	29.94	**<0.001**
**Education (years)**		11.93 ± 3.11	10.86 ± 3.99	10.56 ± 3.94	9.95 ± 3.88	2.16	0.093
**Education Status**	**Elementary**	8 (17.78%)	26 (40.63%)	21 (33.87%)	19 (43.18%)	8.34	0.215
	**High school**	18 (40.00%)	20 (31.25%)	21 (33.87%)	12 (27.27%)		
	**University**	19 (42.22%)	18 (28.13%)	20 (32.26%)	13 (29.55%)		
Occupation status *		38 (84.44%)	28 (43.75%)	32 (51.61%)	14 (31.82%)	27.92	**<0.001**
**Duration of the disease**			88.23 ± 91.03	56.08 ± 63.03	160.86 ± 120.01	26.82	**<0.001**
**Age at onset**			24.25 ± 8.64	31.70 ± 9.95	22.09 ± 6.06	21.56 ^†^	**<0.001**
**Number of** **hospitalizations**			2.11 ± 3.69	0.20 ± 0.70	2.82 ± 4.18	11.40	**<0.001**
**Number of suicide** **attempts**			0.40 ± 1.45	0.19 ± 0.63	0.20 ± 0.58	1.42	0.240
**Tobacco (*n* of users)**		34 (75.56%)	39 (60.94%)	36 (58.06%)	24 (54.54%)	4.81	0.186
**CEDA**			276.75 ± 235.07	113.16 ± 148.77	427.26 ± 295.95	7.47	**0.001**
**HDRS**		4.11 ± 7.39	7.80 ± 8.31	9.70 ± 9.05	N/A	4.69	**0.004**
**YMRS**		N/A	1.68 ± 3.71	N/A	N/A		
**SANS**		N/A	N/A	N/A	21.05 ± 16.70		
**SAPS**		N/A	N/A	N/A	9.23 ± 11.56		
**SF-36**	**Physical function**	25.18 ± 5.25	22.14 ± 6.81	20.47 ± 5.65	20.05 ± 6.11	6.81	**<0.001**
	**Physical Role**	6.76 ± 1.53	6.48 ± 3.21	5.81 ± 1.76	5.33 ± 1.44	3.82	**0.011**
	**Pain**	8.55 ± 2.48	8.36 ± 3.04	7.16 ± 2.29	7.59 ± 3.19	2.94	0.034 **
	**General Health**	16.58 ± 4.65	15.11 ± 4.96	12.83 ± 4.59	13.76 ± 5.08	5.76	**0.001**
	**Vitality ^‡^**	15.00 ± 4.40 15.00 (11.50–18.00)	13.66 ± 4.60 14.00 (10.00–17.75)	11.20 ± 5.03 10.50 (7.00–14.00)	12.05 ± 4.50 12.00 (9.00–15.00)	6.57	**<0.001**
	**Social** **function**	7.78 ± 1.83	6.72 ± 2.28	6.64 ± 1.95	6.59 ± 2.19	3.44	**0.018**
	**Emotional Role**	4.93 ± 1.25	4.23 ± 1.14	4.16 ± 1.21	4.08 ± 1.04	5.43	**0.001**
	**Mental health**	19.98 ± 4.99	17.88 ± 5.67	16.50 ± 5.22	17.56 ± 4.96	3.76	**0.012**
**Number of episodes**	**Total**		5.09 ± 5.28	2.00 ± 1.41		0.78	0.463
	**Manic**		1.41 ± 2.89	-			
	**Mixed**		2.33 ± 2.58	0.50 ± 0.071		0.88	0.422
	**Depressive**		1.52 ± 3.26	1.50 ± 2.12		0.11	0.898

Values are presented as mean ± standard deviation unless otherwise indicated. Categorical variables are presented as n (%). Group comparisons were performed using one-way analysis of variance (ANOVA) or χ^2^ tests, as appropriate. Bonferroni-adjusted post hoc tests were used for pairwise comparisons when homogeneity of variance assumptions were met; Tamhane’s T2 test was used when variances were unequal. HC, healthy controls; BD, bipolar disorder; rMDD, recurrent major depressive disorder; SZ, schizophrenia; CEDA, chlorpromazine-equivalent antipsychotic dose; HDRS, Hamilton Depression Rating Scale; YMRS, Young Mania Rating Scale; SANS, Scale for the Assessment of Negative Symptoms; SAPS, Scale for the Assessment of Positive Symptoms. N/A: Not applicable. ^†^ Analysis performed only among patient groups (BD, rMDD, and SZ). ^‡^ Presented as median (interquartile range) because of non-normal distribution. * Levene’s test indicated unequal variances (*p* < 0.05); Tamhane’s T2 test was used for post hoc pairwise comparisons. ** Although the omnibus ANOVA reached statistical significance, post hoc pairwise comparisons did not yield statistically significant differences between individual groups after correction for multiple comparisons.

**Table 2:** Comparison of neurocognitive performance across study groups.

			HC (*n* = 45)	BD (*n* = 64)	rMDD (*n* = 62)	SZ (*n* = 44)	F/X^2^	*p*
ToM Total *			12.62 ± 2.72	10.92 ± 3.27	10.96 ± 2.65	8.10 ± 3.52	16.46	**<0.001**
ToM Control *			5.64 ± 0.65	5.41 ± 0.95	5.49 ± 0.74	5.10 ± 1.92	1.84	0.141
TMT-A *			38.64 ± 17.97	59.94 ± 33.42	46.20 ± 19.48	72.29 ± 41.67	11.98	**<0.001**
**TMT-B**			91.62 ± 48.11	128.59 ± 72.01	107.66 ± 56.55	141.65 ± 70.51	5.68	**0.001**
DSST *			67.80 ± 17.22	49.73 ± 21.45	55.66 ± 21.72	35.28 ± 17.80	20.32	**<0.001**
**DSST mistakes**			0.07 ± 0.33	0.21 ± 1.03	0.10 ± 0.35	0.07 ± 0.26	0.63	0.599
DSST correct *			65.91 ± 19.87	49.16 ± 22.36	53.87 ± 23.76	35.21 ± 17.84	15.54	**<0.001**
**Digit Span Test**			6.47 ± 1.34	5.94 ± 1.45	5.98 ± 1.49	5.63 ± 1.48	2.56	0.056
Digit Span Test reverse *			3.96 ± 0.93	3.51 ± 1.28	3.64 ± 1.30	3.21 ± 1.25	2.89	**0.036**
**Reaction Time (visual)**			0.34 ± 0.07	0.43 ± 0.15	0.42 ± 0.17	0.55 ± 0.28	10.29	**<0.001**
**Reaction Time (auditory)**			0.32 ± 0.09	0.38 ± 0.14	0.37 ± 0.09	0.48 ± 0.20	10.11	**<0.001**
**AMIPB-A**		**1st trial**	49.84 ± 10.33	40.48 ± 13.68	41.79 ± 13.34	32.95 ± 11.63	13.46	**<0.001**
		**2nd trial**	67.89 ± 19.88 71.00 (11.50–18.00)	51.17 ± 19.74 50.00 (37.00–67.00)	50.85 ± 21.99 51.00 (36.00–66.00)	35.56 ± 19.09 35.00 (22.00–51.00)	18.61	**<0.001**
		**True**	63.62 ± 19.22 70.00 (53.00–76.50)	46.84 ± 20.89 49.00 (30.00–62.00)	47.98 ± 21.86 48.00 (33.50–62.50)	29.40 ± 18.43 28.00 (11.00–40.00)	20.75	**<0.001**
		**False**	4.22 ± 3.80	4.33 ± 4.99	4.34 ± 3.44	6.09 ± 11.05	0.95	0.418
		Correction *	1.67 ± 1.43	1.56 ± 2.46	1.15 ± 1.67	0.63 ± 1.13	3.12	**0.027**
**AMIPB-B**		**1st trial**	59.84 ± 19.60	42.73 ± 14.02	45.33 ± 12.70	34.37 ± 13.59	16.73	**<0.001**
		**2nd trial**	59.84 ± 19.60	42.41 ± 21.02	45.92 ± 18.28	31.14 ± 19.69	16.03	**<0.001**
		**True**	58.24 ± 19.27	39.56 ± 21.56	43.97 ± 18.75	30.19 ± 20.41	15.17	**<0.001**
		**False**	1.56 ± 2.15	1.35 ± 2.70	1.93 ± 2.79	2.30 ± 7.62	0.53	0.665
		**Correction**	0.84 ± 1.06	0.50 ± 0.99	0.46 ± 0.77	0.35 ± 0.69	2.61	0.052
**Stroop Test**	**1**	Duration *	8.63 ± 2.08	10.73 ± 3.95	10.44 ± 2.84	12.38 ± 5.11	7.89	**<0.001**
		**Mistake**	0.00 ± 0.00	0.11 ± 0.48	0.00 ± 0.00	0.00 ± 0.00	2.65	0.050
		**Correction**	0.00 ± 0.00	0.05 ± 0.22	0.07 ± 0.31	0.02 ± 0.15	0.89	0.447
	**2**	**Duration**	9.33 ± 3.20	12.17 ± 5.05	12.49 ± 7.21	13.56 ± 5.87	4.60	**0.004**
		**Mistake**	0.09 ± 0.42	0.14 ± 0.78	0.02 ± 0.13	0.07 ± 0.34	0.68	0.567
		**Correction**	0.07 ± 0.33	0.08 ± 0.41	0.13 ± 0.50	0.19 ± 0.55	0.71	0.548
	**3**	Duration *	12.45 ± 3.32	16.37 ± 5.83	13.93 ± 3.97	19.75 ± 8.41	14.76	**<0.001**
		**Mistake**	0.04 ± 0.21	0.35 ± 1.31	0.20 ± 0.83	0.29 ± 0.81	1.07	0.363
		Correction *	0.22 ± 0.47	0.35 ± 0.63	0.20 ± 0.54	0.57 ± 0.99	3.05	**0.030**
	**4**	Duration *	16.36 ± 5.34	23.78 ± 13.94	20.21 ± 7.33	28.04 ± 15.45	8.83	**<0.001**
		**Mistake**	0.13 ± 0.46	0.40 ± 1.26	0.13 ± 0.39	0.86 ± 3.59	1.71	0.166
		Correction *	0.13 ± 0.34	0.37 ± 0.68	0.36 ± 0.63	0.81 ± 0.94	7.57	**<0.001**
	**5**	Duration *	22.92 ± 7.31	33.15 ± 17.54	29.03 ± 8.89	42.45 ± 22.58	13.07	**<0.001**
		**Mistake**	0.80 ± 1.31	1.48 ± 3.60	1.11 ± 1.79	2.14 ± 4.28	1.69	0.170
		Correction *	0.87 ± 1.16	1.18 ± 1.65	1.11 ± 1.31	1.86 ± 1.57	3.89	**0.010**

Values are presented as mean ± standard deviation unless otherwise indicated. Group comparisons were performed using one-way analysis of variance (ANOVA) or Kruskal–Wallis tests, as appropriate. Bonferroni-adjusted post hoc tests were used when homogeneity of variances was met, whereas Tamhane’s T2 test was used when variances were unequal. Higher scores on TMT, reaction time, and Stroop duration measures indicate poorer performance. TMT = Trail Making Test; ToM = Theory of Mind; DSST = Digit Symbol Substitution Test; AMIPB = Adult Memory and Information Processing Battery. * Levene’s test indicated unequal variances (*p* < 0.05); Tamhane’s T2 test was used for post hoc pairwise comparisons.

**Table 3:** Comparison of metabolic variables across study groups.

	HC (*n* = 45)	BD (*n* = 64)	rMDD (*n* = 62)	SZ (*n* = 44)	F/Z/X^2^	*p*
Waist Circumference *	89.38 ± 8.12	99.64 ± 12.36	92.92 ± 7.90	96.09 ± 10.99	9.99	**<0.001**
BMI *	24.51 ± 3.86	28.09 ± 5.28	25.58 ± 3.93	26.52 ± 5.67	5.56	**0.001**
**LDL**	101.30 ± 32.52	107.05 ± 34.66	121.25 ± 31.74	109.57 ± 37.35	3.31	**0.021**
**HDL**	46.82 ± 11.04	46.88 ± 12.49	47.43 ± 13.71	42.27 ± 17.46	1.41	0.240
**Total Cholesterol**	174.86 ± 35.91	179.98 ± 40.23	196.42 ± 35.10	184.86 ± 37.47	3.29	**0.022**
**Triglyceride**	131.77 ± 65.07	129.86 ± 74.01	144.10 ± 104.41	159.37 ± 120.61	1.03	0.379
Fasting glucose *	88.78 ± 8.97	91.49 ± 8.97	88.86 ± 6.62	91.88 ± 7.49	2.31	0.078
HbA1c *	5.28 ± 0.49	5.27 ± 0.46	5.31 ± 0.33	5.67 ± 1.35	3.28	**0.022**

Values are presented as mean ± standard deviation. Group comparisons were performed using one-way analysis of variance (ANOVA). Bonferroni-adjusted post hoc tests were used when homogeneity of variances was met, whereas Tamhane’s T2 test was used when variances were unequal. BMI, body mass index; LDL, low-density lipoprotein cholesterol; HDL, high-density lipoprotein cholesterol; HbA1c, glycated hemoglobin. * Levene’s test indicated unequal variances (*p* < 0.05); Tamhane’s T2 test was used for post hoc pairwise comparisons.

**Table 4:** Pearson correlations between HbA1c levels and neurocognitive measures across study groups.

	HC	BD	rMDD	SZ
**TMT-A**	0.24	0.39 *	0.23	0.06
**TMT-B**	0.29	0.58 **	0.27 *	0.04
**AMIPB-A1**	−0.32 *	−0.33 **	−0.16	−0.14
**AMIPB-A2**	−0.39 *	−0.33 **	−0.23	0.01
**AMIPB-B1**	−0.41 **	−0.33 **	−0.22	−0.12
**AMIPB-B2**	−0.38 *	−0.36 **	−0.23	−0.08
**ToM total**	−0.25	−0.29 *	−0.19	0.16
**DSST**	−0.54 **	−0.39 **	−0.22	−0.05
**Digit Span Test**	−0.31 *	−0.27 *	−0.19	−0.07
**Digit Span -Reverse**	−0.22	−0.35 *	−0.20	−0.16
**ST-1 duration**	0.41 **	0.37 **	0.07	0.10
**ST-1 correction**	-	0.39 **	0.11	−0.01
**ST-2 duration**	0.68 **	0.54 **	−0.05	0.14
**ST-2 mistake**	0.33 *	0.20	−0.12	−0.10
**ST-2 correction**	0.79 **	0.63 **	0.06	0.03
**ST-3 duration**	0.57 **	0.28 *	−0.10	0.01
**ST-3 mistake**	0.03	0.47 **	0.15	−0.02
**ST-4 duration**	0.53 **	0.44 **	−0.01	0.18
**ST-4 mistake**	−0.06	0.67 **	0.19	−0.03
**ST-5 duration**	0.35 *	0.30 *	0.05	0.07
**ST-5 mistake**	0.13	0.43 **	0.25	0.01

Pearson correlation coefficients (r) are presented. * *p* < 0.05; ** *p* < 0.01. HbA1c, glycated hemoglobin; TMT, Trail Making Test; AMIPB, Adult Memory and Information Processing Battery; ToM, Theory of Mind; DSST, Digit Symbol Substitution Test; ST, Stroop Test. Additional abbreviations are provided in Table 2.

**Table 5:** Linear regression analyses examining the association between HbA1c levels and neurocognitive performance.

Neurocognitive Test Score	Group	Unstandardized Coefficients		Standardized Coefficients	t	*p*
		B	Std Error	β		
**TMT-A**	BD^1^	28.37	8.54	0.39	3.32	0.002
**TMT-B**	BD^2^	89.70	16.35	0.58	5.49	**<0.001**
**TMT-B**	MDD^3^	46.89	21.69	0.27	2.16	0.035
**AMIPB-A1**	HC^4^	−6.76	3.14	−0.32	−2.16	0.037
**AMIPB-A1**	BD^5^	−9.81	3.59	−0.33	−2.73	0.008
**AMIPB-B1**	HC^6^	−8.50	2.94	−0.41	−2.89	0.006
**AMIPB-B1**	BD^7^	−10.11	3.67	−0.33	−2.75	0.008
**AMIPB-B2**	HC^8^	−15.20	5.78	−0.38	−2.63	0.012
**AMIPB-B2**	BD^9^	−16.53	5.44	−0.36	−3.04	0.003
**ToM total**	BD^10^	−2.00	0.89	−0.29	−2.25	0.028
**DSST**	HC^11^	−18.86	4.60	−0.54	−4.10	**<0.001**
**DSST**	BD^12^	−17.86	5.50	−0.38	−3.25	0.002
**Digit-Span Test**	HC^13^	−0.87	0.41	−0.31	−2.13	0.039
**Digit-Span Test**	BD^14^	−0.85	0.39	−0.27	−2.19	0.032
**Digit-Span Test reverse**	BD^15^	−0.98	0.33	−0.35	−2.95	0.005
**ST-1 duration**	HC^16^	1.76	0.61	0.41	2.91	0.006
**ST-1 duration**	BD^17^	3.16	1.02	0.37	3.10	0.003
**ST-2 duration**	HC^18^	4.45	0.75	0.68	5.93	**<0.001**
**ST-2 duration**	BD^19^	5.94	1.18	0.54	5.03	**<0.001**
**ST-3 duration**	HC^20^	3.94	0.87	0.57	4.55	**<0.001**
**ST-3 duration**	BD^21^	3.48	1.56	0.28	2.24	0.029
**ST-4 duration**	HC^22^	5.88	1.44	0.53	4.09	**<0.001**
**ST-4 duration**	BD^23^	13.27	3.47	0.44	3.82	**<0.001**
**ST-5 duration**	HC^24^	5.36	2.19	0.35	2.45	0.019
**ST-5 duration**	BD^25^	11.09	4.66	0.30	2.38	0.021

Linear regression analyses were conducted with HbA1c as the independent variable. Only significant results are presented. Values represent unstandardized (B) and standardized (β) coefficients, standard error (SE), t values, and *p* values. TMT = Trail Making Test; ToM = Theory of Mind; DSST = Digit Symbol Substitution Test; AMIPB = Adult Memory and Information Processing Battery; ST = Stroop Test.

**Table 6:** Model fit statistics and approximate effect sizes for hierarchical regression analyses examining the association between HbA1c and neurocognitive performance.

Outcome	Group	Adjusted R^2^	F	Approximate Cohen’s f^2^
**TMT-A**	BD	0.14	11.04	0.163
**TMT-B**	BD	0.33	30.10	0.493
**TMT-B**	rMDD	0.06	4.67	0.064
**AMIPB-A1**	HC	0.08	7.12	0.087
**AMIPB-A1**	BD	0.10	7.37	0.111
**AMIPB-B1**	HC	0.15	8.36	0.176
**AMIPB-B1**	BD	0.10	7.58	0.111
**AMIPB-B2**	HC	0.12	6.91	0.136
**AMIPB-B2**	BD	0.12	9.24	0.136
**ToM total**	BD	0.07	5.07	0.075
**DSST**	HC	0.27	16.81	0.370
**DSST**	BD	0.13	10.55	0.149
**Digit Span**	HC	0.08	10.55 *	0.087
**Digit Span**	BD	0.06	4.80	0.064
**Digit Span reverse**	BD	0.11	8.70	0.124
**ST-1 duration**	HC	0.15	8.44	0.176
**ST-1 duration**	BD	0.12	9.61	0.136
**ST-2 duration**	HC	0.44	9.61	0.786
**ST-2 duration**	BD	0.28	25.31	0.389
**ST-3 duration**	HC	0.31	20.68	0.449
**ST-3 duration**	BD	0.06	5.00	0.064
**ST-4 duration**	HC	0.27	16.73	0.370
**ST-4 duration**	BD	0.18	14.61	0.220
**ST-5 duration**	HC	0.10	6.00	0.111
**ST-5 duration**	BD	0.07	5.65	0.075

Adjusted R^2^ and F values reflect the final hierarchical regression model for each outcome (see Statistical Analysis for model-building steps); no significant regression effects were observed in the SZ group (see Results). Approximate Cohen’s f^2^ was calculated as f^2^ = R^2^/(1 − R^2^) using the adjusted R^2^ reported for each model, as a standardized index of overall model effect size (small ≈ 0.02, medium ≈ 0.15, large ≈ 0.35, per Cohen, 1988). See Table 5 for individual regression coefficients (B, SE, β, t, *p*). TMT = Trail Making Test; ToM = Theory of Mind; DSST = Digit Symbol Substitution Test; AMIPB = Adult Memory and Information Processing Battery; ST = Stroop Test. * The F value for Digit Span in the HC group is coincidentally identical to that of DSST in the BD group (F = 10.55); this reflects the independently computed statistics for each model and is not a duplication error.

## Data Availability

The datasets and materials in this study are available on request from the corresponding author.

## Supplementary Materials

The following supporting information can be downloaded at: https://www.mdpi.com/article/10.3390/brainsci16070770/s1, Table S1: Psychotropic Medication Summary by Diagnostic Group.
