# Supplementary material for: HbA1c and Cognitive Functioning Across Bipolar Disorder, Recurrent Major Depressive Disorder, and Schizophrenia: Findings from a Non-Diabetic Sample
Source: Brain Sci. 2026 Jul 22;16(7):770. doi: 10.3390/brainsci16070770 (PMC13406456; doi:10.3390/brainsci16070770)
Supplement: Supplementary file 1 [file brainsci-16-00770-s001.zip › brainsci-4405253-supplementary.pdf]

Table S1. Psychotropic Medication Summary by Diagnostic Group

	HC	BD	rMDD	SZ
Antipsychotic dose (CEDA, mg/day), n (Mean ± SD)	0	44 (276.75 ± 235.07)	10 (113.16 ± 148.77)	42 (427.26 ± 295.95)
Lithium level (mEq/L), n (Mean ± SD)	0	14 (0.47 ± 0.22)	1 (0.85)	0
Valproic acid level (µg/mL), n (Mean ± SD)	0	30 (64.17 ± 21.28)	1 (81.20)	1 (31.40)

Abbreviations: HC, healthy controls; BD, bipolar disorder; rMDD, recurrent major depressive disorder; SZ, schizophrenia; CEDA, chlorpromazine-equivalent antipsychotic dose; SD, standard deviation. Lithium and valproic acid levels reflect therapeutic drug monitoring values available for a subset of treated patients.
